# Knowledge growth and development: internet of things (IoT) research, 2006–2018

**DOI:** 10.1016/j.heliyon.2019.e02264

**Published:** 2019-08-28

**Authors:** M. Dachyar, Teuku Yuri M. Zagloel, L. Ranjaliba Saragih

**Affiliations:** Department of Industrial Engineering, Universitas Indonesia, Depok 16424, Indonesia

**Keywords:** Information science, Scientometrics, 4th Industrial revolution, Internet of things, Knowledge growth, Scopus, IOT

## Abstract

The term "Internet of Things" first appeared in publication paper since 2006, describing the paradigm of evolution concept that brought about by the presence of internet technology (Vermesan and Friess, 2015) which is very important in contemporary circumstances. This study conducted an in-depth analysis of the research material written on 26420 papers which focused on the published Internet of Things (IoT) research, starting from the firstly year IoT keyword appeared in 2006 until 2018. The selected paper is a combination of various disciplines and publications which are all indexed by Scopus wherein the article discusses IoT. IoT articles are classified using key attributes in sequence: the methodology used, general knowledge and applied concepts, and various general exploration topics. By using the Scientometrics method, this method will group the overall terms that appear frequently from the Scopus paper database according to keywords, titles, and abstracts. The resulting data is then studied to understand and distinguish trends that occur in the time span along with the general characteristics of the paper, in the mathematics visual scheme. All various issues that are considered in the paper's methodology selection, their studied and services innovations, and continuing discoveries on the characteristics, concepts, and processes applied to IoT success. Although it only involves scopus indexed paper, this study found a remarkable increase in the number of articles on IoT in each category of the paper. This study also reveals the direction of the regular discipline of knowledge. The use of the Scientometrics method makes the analysis able to focus on the movement of characteristics and IoT themes to researcher's direction that has not found at this time, as a comprehensive guide to further research and industry strategy that is more directed on concepts that support the 4th industrial revolution.

## 1 Introduction

**Fig. 1:**
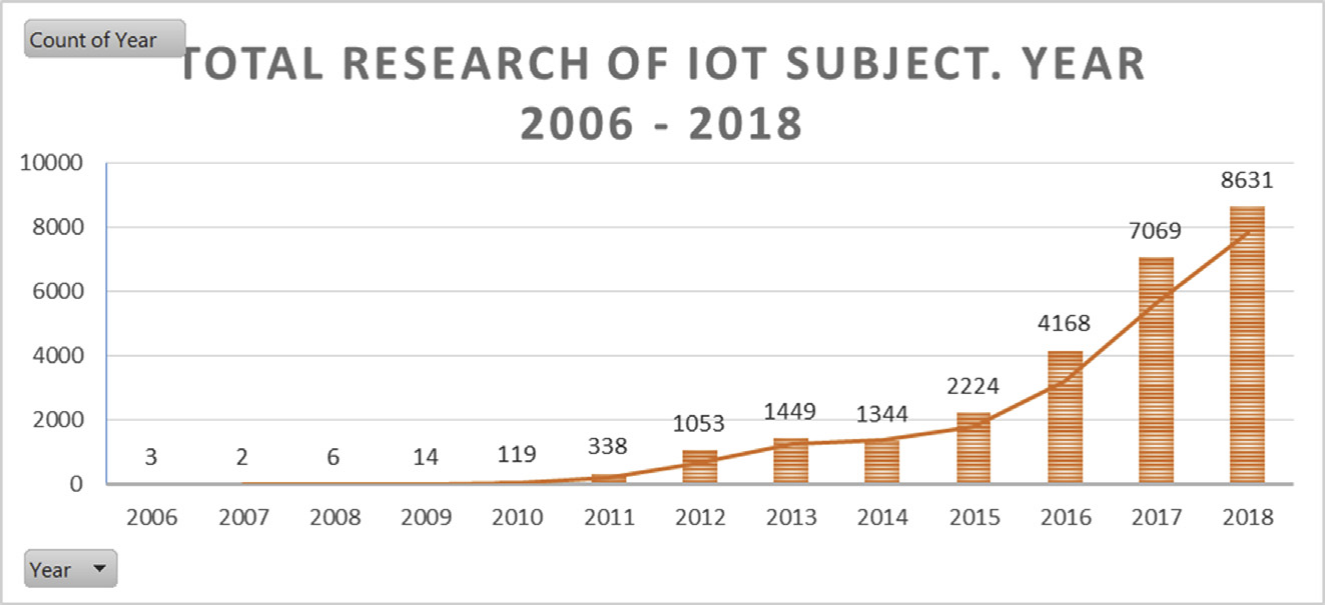
Number of the IoT research in 2006–2018.

The entire study in the paper between 2006 and 2018 carries a record of 8510 journal papers and 16775 conference proceeding papers that discuss the Internet of Things. The article Internet of Things starts from the following three conference papers (Adelmann et al., 2006; Bernard, 2006; Rammig et al., 2006) published in 2006 even though there is only one paper that explicitly uses the internet of things sentence in its title, while the other two mention it in the abstract. Then there were only two conference papers in 2007 (Muenchen et al., 2007; Thiesse et al., 2007), and became increased to six papers in 2008 (Elrharbi and Pépin, 2008; Frenken and Spiess, 2008; Grønbæk and Telenor, 2008; Kong et al., 2008; Nyman et al., 2008; Wu, 2008) until the time of writing this paper at the end of 2018 there were a total of 25285 published papers. Developments in the early three years of the Internet of Things research, all types of paper documents in the form of conference papers that contain technical applications in connecting real-world objects to virtual information using computing system mobility, for example, mobile phones or handheld PDAs, by instilling an introduction to reading algorithms barcode or RFID to start IoT for the future better (Adelmann et al., 2006) which later developed into a variety of big things to date. The percentage of occurrence in the initial three years is 6.2 × 10-4% can be considered as a very less significant value in the development of knowledge. The graph data in Fig. 1 indicate IoT began to be interested in being investigated since 2010 with a large number of researches (total 119) then continuing with developments multiplying times each year until this paper was made.

The research area of the Internet of Things in recent years has experienced growth and development in an interdisciplinary manner. Various papers are written very massive, and reach various concepts and fields of knowledge ranging from technology, applied engineering, economics, business, strategy, industry, management, etc. This becomes a confusion in understanding the direction of the development of the IoT knowledge. Besides this, IoT brings many dimensions of disruptive to many things for humans and nature such as physical disruption in terms of work, cyberspace disruptions that make human work deprived and faced with various new complexities when carrying out their routine activities and tasks, disruption of data through mastery of information generated by big data which causes many opportunities for new knowledge to emerge and new creative intelligent environments greatly changes the current conditions (Ammirato et al., 2018), including interfering with many everyday business processes both the specifications of people, their abilities and routine tasks, which are directed towards benefits characterized by high speed and interconnection.

Most of the research is written on the applied scale of technology in using IoT, run as something that facilitates human life in certain fields, including how IoT helps improve business capabilities, and recent research analyzes how data obtained from IoT devices can benefit various aspects. But looking at the disruptive level that spreads across all industrial areas (so-called revolution) and becomes a topic that is widely discussed in the future through the 4th industrial revolution scheme. It is a gap to write a science thinking flow as a foundation that addresses this IoT growth and development that can be used by researchers, developers and industries. The direction of the development of IoT is expected to be easier to understand, how the character and habits of the problem occur, and various methods and tools used by researchers related to the knowledge domain and industry.

The purpose of this paper is to explore the theoretical core of the IoT. This paper discusses the research questions as follows:

RQ1. How does the core knowledge of the field of the Internet of Things evolve through time?

RQ2. What are the most influential industries in the Internet of Things knowledge field?

RQ3. What are the substance methods or tools used in the Internet of Things research?

The research approach is an in-depth literature analysis using Scientometrics technique with a comprehensive information visualization technology in the entire journal paper and conference paper mentioned above. In order to process large-scale documents, we need a tool for software and data visualization technology that is capable of handling large amounts of scientific literature data. VOSviewer is used which has a special function for mining text that can be used to build, visualize and explore bibliometric maps of keywords taken from a large collection of scientific literature that presented in various ways, through keyword repeat mapping, which can provide visualization of results through various bibliometric indicators (Jin and Ji, 2018; Sajovic et al., 2018; Youngblood and Lahti, 2018) by interpreting a mathematic scheme in the linked-circular theme and progressive average year, so that research and trends in certain fields can be more clearly demonstrated. In addition to contributing to the more clearly found portrayals of various fields, an additional qualitative analysis was also conducted to bridge the gap between the IoT and various findings of scientific disciplines related to research content.

The structure of the paper is communicated as follows in sequence: starting with the background, this paper presents IoT research to the present, identifying research gaps and motivations to overcome them through answers to defined research questions. The methodology section explains the sequence of stages of obtaining material and methods, analysis of repetition of keywords and clusters, classification of data, and analysis of results. The following subsections show IoT evolution followed by presentations of the five largest fundamental themes of the group area and related knowledge disciplines, to further identify the state-of-the-art field of the IoT. The end result is a conclusion as an outlook from the IoT and the future of the work area. The writing of the sub-section of the paper further shows the evolution of IoT in the disciplinary group of knowledge and a description of the overall main character in the field of industry, then identifying what details the various methods and tools in implementing the concept of the IoT. We also report an approach to the thinking process of scientists in formulating their IoT research as a sequential flow of state-of-the-art concepts. The end result is a conclusion as an outlook from the IoT and the future of the work area.

## 2 Methodology

This study answers the research questions stated in the introduction by applying quantitative literature review using Scientometrics Analysis based on the bibliometric techniques on keywords, abstracts, and titles.

In order to get a comprehensive dataset, our data sources are generated from the results of Scopus database queries, with an advanced search using the "IoT" AND "Internet of Things" search phrases that found in Titles, Abstracts and Paper Keywords.

To make the dataset obtained relevantly and to avoid invalid documents, a set of criteria needs to be defined. Following are the methods used to include/remove documents from the analysis dataset:

• Only admit paper indexed by Scopus, with document type: *conference paper*, *article* and *article in press*; Emit other types of documents such as a *book, chapter book, editorial, letter, note, review,* and *short survey*;
• Admit papers written in English;
• Eliminate duplication, and data formatted incorrectly on each dataset item;
• Admit paper from the entire year and publisher;

The study uses a VOSviewer software tool based on co-citation to produce any term map based on co-occurrence data that is processed based on the title, keyword and abstract from the dataset provided, where the conditions specified are terms with the number of occurrences >10 times (configuration set). This tool displays an analysis of bibliometric maps of paper that are processed in detail. Then the research uses the VOSviewer software tool to create a paper map based on co-citation to produce any term map based on co-occurrence data that is processed based on the title, keyword, and abstract of the entered dataset, where the conditions specified are in with the number of occurrences >10 times. This tool displays the bibliometric map analysis of the entire paper processed in detail (van Eck and Waltman, 2010) and as an analytical method for mapping science that is able to identify knowledge of useful terms from data, networks, and maps (Sajovic et al., 2018).

**Fig. 2:**
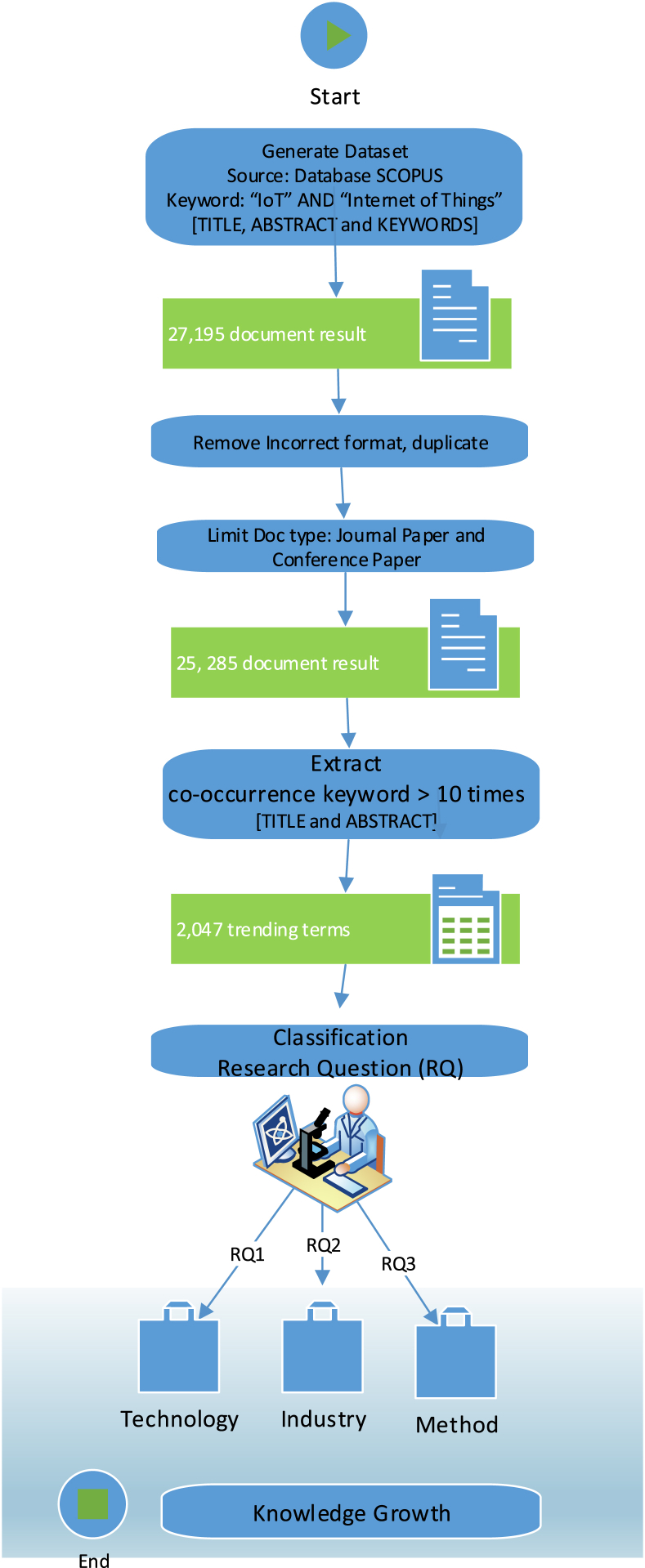
Research methodology.

The results of this bibliometric analysis result in trending items in the form of research terms that run within the span of the research marked by the number of occurrences and average years of publication, this answers the research question one about how does the core knowledge of the field of the Internet of Things evolve through time (RQ1); the results are then processed to answer the research question two about what are the most influential industries in the Internet of Things knowledge field (RQ2) in order to produce any industry influenced by the Internet of Things. Fig. 2 shows the research methodology.

At the end of Fig. 2, data segregation is carried out in order to have benefits in answering research questions. The core knowledge that moves the IoT research period will be related to certain technological fields that are automatically marked based on the labels that appear according to the tools used. Segregation in terms of industry is the most influential through the results of manually defining all data found with label-to-industry mapping. Segregation to get the method used in the article, obtained by redefining certain attributes of each industry in Scopus database search, through various articles according to each industry obtained then examined about what methods and tools are used by researchers (manual and automatic segregation).

## 3 Study area

**Fig. 3:**
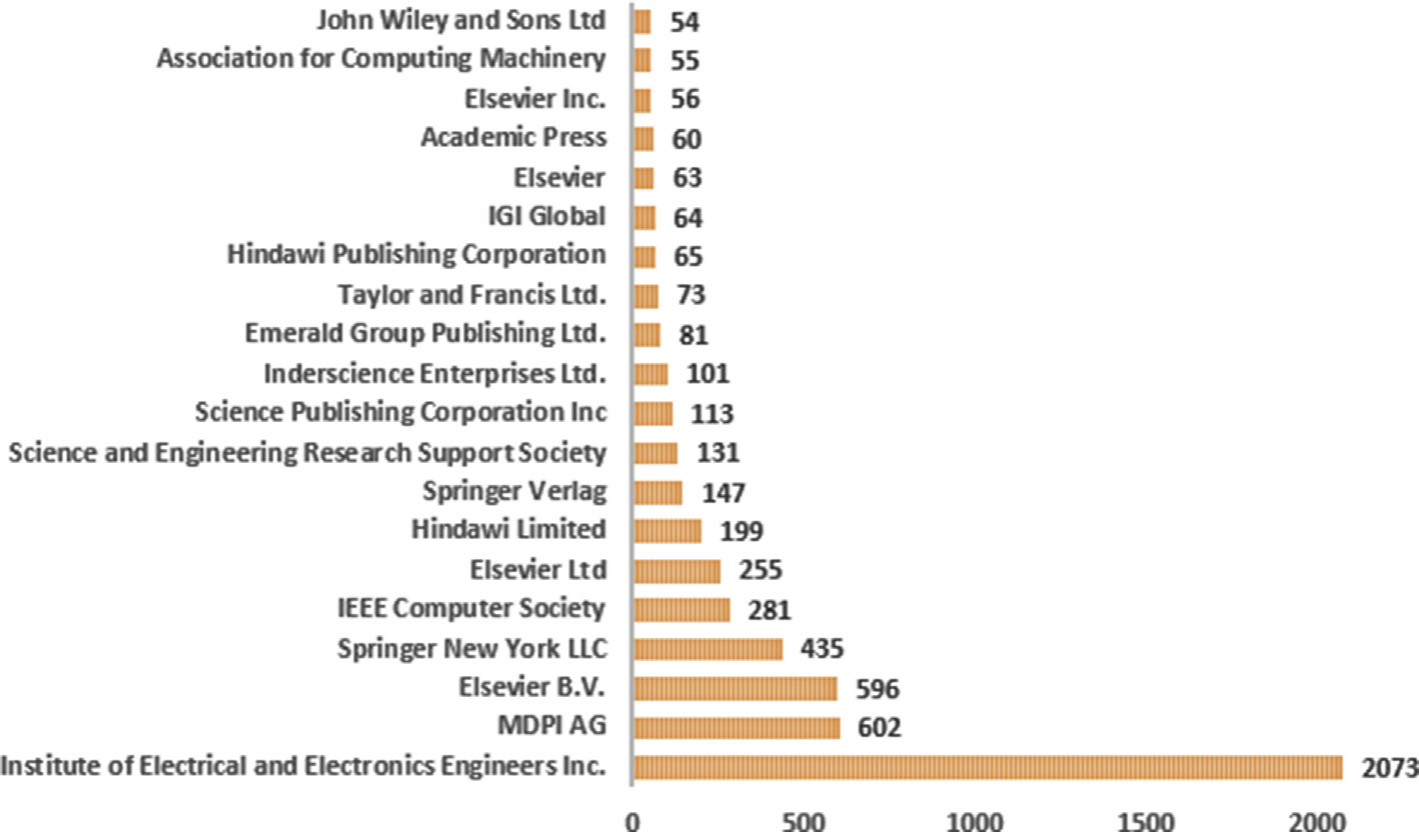
Top 20 publisher of IoT's paper.

Scopus has indexed 469 publishers around the world who have published papers that write a variety of knowledge about IoT, in Fig. 3, there are 20 top publishers, including 3 of the most active ones, namely IEEE, MDPI AG and Elsevier B.V. IEEE publishers produce most research publications, and they furthermore have issued 80 standards, 45 ongoing projects, including the most famous is IEEE P2413 (Draft Standard for the Architectural Framework for the IoT), and also has its own research Working Group that focuses on the field of IoT (IEEE Standards Association, 2018), this is different from MDPI AG and Elsevier B.V which only play a role in publishing journal papers and IoT conference papers.

The topic of IoT research involves various industries, which are considered disruptive and threats to various sectors because of the presence of internet technology that confirms changes in business patterns, humans in work, communication, transportation, factories, etc. that replace human functions, or significantly change values corporate chain through defining various new business models. It is increasingly scattered and increasing in quantity, this is in line with the development of knowledge which is characterized by irregular developments.

**Fig. 4:**
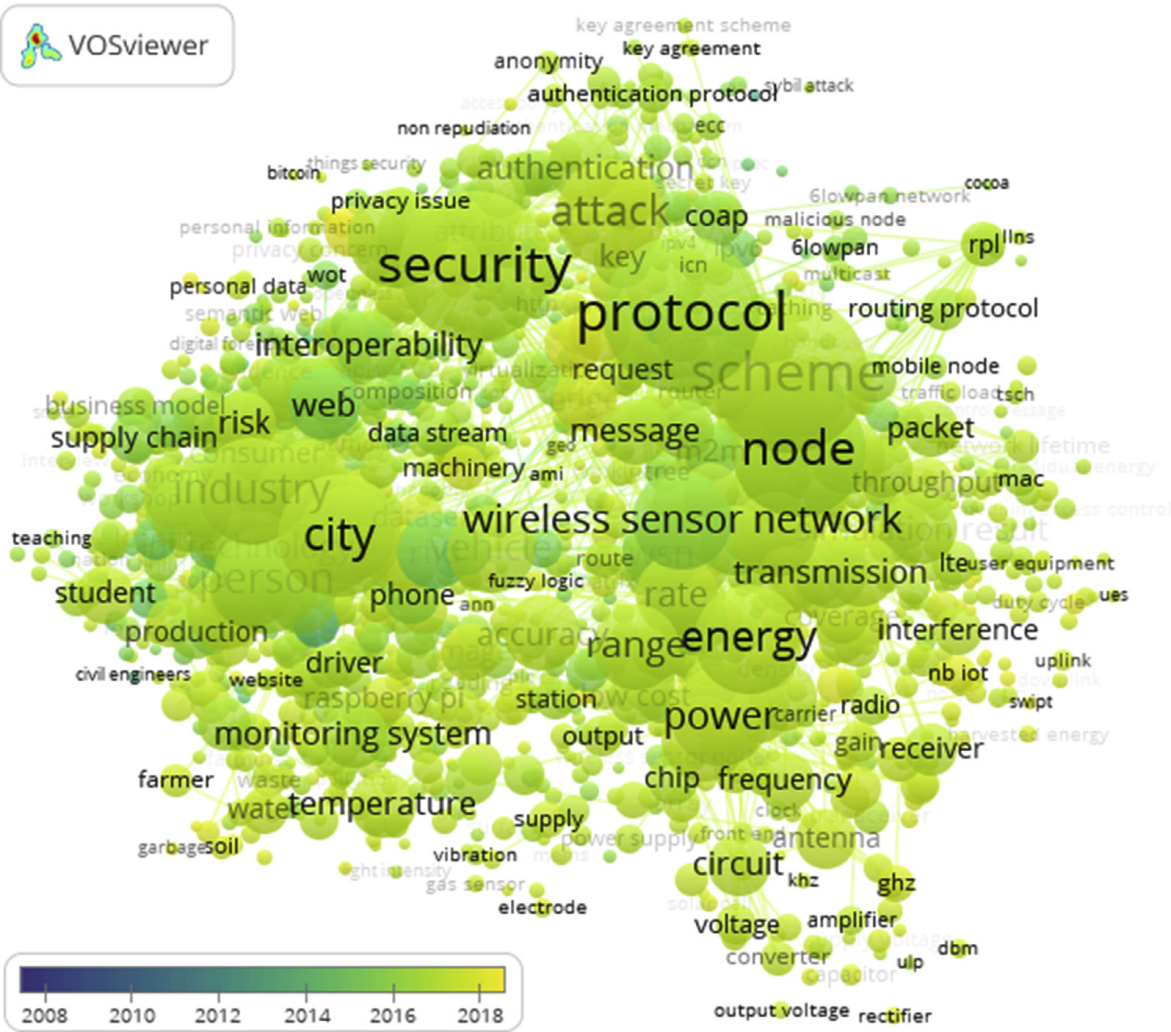
The entire term in the IoT research paper in the period 2006–2018.

Through VOSviewer tools we find various studies of researchers who move according to the times and conditions that are considered the most hits in time, so that it is expected to map a very large and complex research trend so that models can show the development of successive research and to be able to see what IoT potential that is in the future. Through Fig. 4, the pattern of analysis of research trends from 2006 to 2018 is shown with different years colors, the occurrence increasing of research terms will be displayed with a larger diameter circle size.

This research we classify various industries which are considered very intensive involving IoT in their business model. The number of industries found is in line with the emergence of research attention in the industrial sector, whether driven by industry needs or researchers' proposals due to the suitability of IoT technical application to related industries. These finding industries can be categorized as mature in applying the 4th industrial revolution concept in their operation where IoT is one of the key elements.

Labels or terms that are found are presented as a combination of the words and sentences that are most widely used by researchers in the period 2006–2018 which recur more than 10 times (according to the configuration set) in the title, keyword, and abstract of all existing research. Total repetition is calculated in the Occurrence column, which displays information on how many terms appear in all the research papers. Average score explains the average of all years of publication found in various years, this indicates the weight of the year distribution which is the quantitative average of the term emerged.

All terms involving industry are displayed, excluded from the telecommunications and ICT industry because both are considered not influenced by IoT and are even the basic industry providers of IoT products themselves (such as vendor service connectivity and various sensor devices as IoT solutions). This study found various industrial sectors that involved or were affected by IoT intensively as shown in Table 1.

**Table 1:** Industrial sector, appearance and year of publication.

Label/Term	weight <Occur-rences>	score<Avg. pub.year>	Industry Sector
logistic	420	2014.8	All Industry
agricultural product	107	2014.8	Agriculture
iot industry	57	2015.3	All Industry
supply chain management	126	2015.4	All Industry
agricultural internet	33	2015.5	Agriculture
agricultural production	54	2015.5	Agriculture
decision support	54	2015.7	All Industry
intelligent transportation system	173	2015.7	Public Service
electric vehicle	96	2015.8	Electronics
production process	100	2015.9	Manufacturing
automotive industry	34	2015.9	Manufacturing
campus	128	2016.1	Public Service
bus	222	2016.1	Manufacturing
mining	392	2016.2	Mining
manufacturing industry	73	2016.2	Manufacturing
agriculture	625	2016.3	Agriculture
school	146	2016.5	Public Service
manufacturing	511	2016.5	Manufacturing
health care	298	2016.5	Health
smart industry	36	2016.5	Manufacturing
robot	466	2016.5	Electronics
education	327	2016.5	Public Service
electronic	408	2016.5	Electronics
industrial process	53	2016.5	Manufacturing
hospital	328	2016.5	Health
industrial control system	52	2016.6	Manufacturing
electricity	144	2016.6	Energy
city	3365	2016.6	Public Service
farm	197	2016.7	Agriculture
electronics	74	2016.7	Electronics
healthcare	875	2016.7	Health
factory	212	2016.7	Manufacturing
production line	52	2016.7	Manufacturing
traffic management	95	2016.7	Public Service
microcontroller	430	2016.8	Electronics
battery	850	2016.8	Energy
transportation system	102	2016.8	Public Service
healthcare service	171	2016.8	Health
plant	330	2016.8	Agriculture
farming	141	2016.8	Agriculture
precision agriculture	89	2016.8	Agriculture
healthcare system	292	2016.8	Health
manufacturing system	123	2016.9	Manufacturing
gas	114	2016.9	Energy
water	508	2016.9	Public Service
production system	119	2017.0	Manufacturing
medicine	151	2017.0	Health
medical data	59	2017.0	Health
wearable	209	2017.0	Electronics
smart manufacturing	99	2017.0	Manufacturing
gas sensor	74	2017.0	Energy
farmer	271	2017.1	Agriculture
smart factory	203	2017.1	Manufacturing
solar energy	36	2017.1	Energy
irrigation system	89	2017.1	Agriculture
healthcare industry	59	2017.2	Health
smart agriculture	76	2017.2	Agriculture
machinery	447	2017.3	Manufacturing

## 4 Results & discussion

Data derived from Table 1 is further processed to categorize paper related to certain industrial sectors. Found seven industries affected by the IoT starting from the most to the least influential as follows Manufacturing, Agriculture, Public Service, Health, Electronics, Energy, and Mining.

### 4.1 Growth and development of the IoT industry

#### 4.1.1 Industry 1: manufacturing

**Fig. 5:**
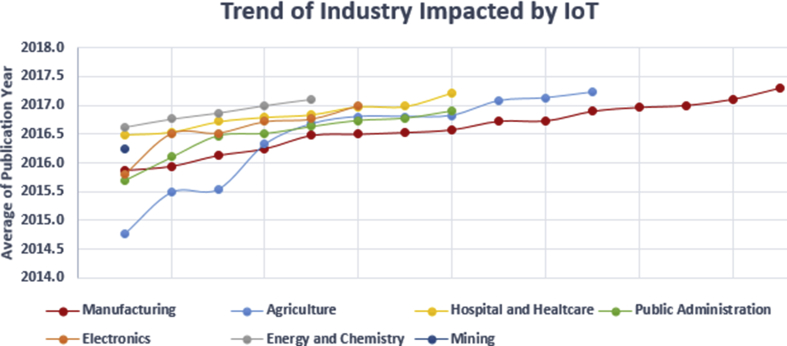
Seven industries affected by IoT.

In this study, IoT looks very influential in the manufacturing industry sector, as shown in Fig. 5, research activities in this field are very intensive and always high throughout the year, this is in line with the 4th industrial revolution that is running throughout the world. Starting from the German government which initiated the term Industrie 4.0 in 2011 in the manufacturing industry sector using the term IIoT (Industrial IoT) through the 4th industrial revolution, which strongly emphasized IoT integration into manufacturing operations and communication between many objects (Kiel et al., 2017). This characterizes the digital connections of industrial manufacturing processes that produce fully intelligent, connected and autonomous plants.

#### 4.1.2 Industry 2: agriculture

The second industry sector that is most influenced by IoT is the agricultural sector where the IoT has recently been applied in agrarian countries, as stated by (Mushtaq, 2018; Srilakshmi et al., 2018) this is driven because IoT contributes significantly to socio-economic growth, Increasing Productivity, Reducing Costs and Optimizing time for farmers in particular, and also this sector produces a basic need that has a great effect on a country in general where its development is strongly influenced by how is the government's strategy (Saragih et al., 2018).

#### 4.1.3 Industry 3: public service

Due to IoT technology ability to connect many services through the internet with the ability of sensor devices and monitoring to be spread everywhere, many researchers are implementing their IoT applications in the sector of intelligent Public Service industry (Hoon et al., 2013; Díaz-díaz, Muñoz and Pérez-gonzález, 2017; Trilles et al., 2017), those are found in this study included intelligent transportation system, smart campus, smart school, general education, city, traffic management, transportation system and water.

#### 4.1.4 Industry 4: electronics

The electronic industry sector which is much influenced by the presence of IoT, all those found are electric vehicles, robots, electronics, electronics, microcontrollers, and wearables. Actually, these findings are product technologies that have long existed, but through IoT now developed with a variety of new capabilities, such as being able to have sensors on the environment, then be able to connect and exchange information with each other through an internet connection.

#### 4.1.5 Industry 5: health

This health industry sector is one of the biggest influenced by IoT, the sector is a new IoT product in health services which includes labels found in it such as Health care, hospital, healthcare, healthcare service, healthcare system, medicine, medical data, healthcare industry. Services that involve IoT such as health condition sensors and historical records that study a person's health condition with qualitative analysis, as well as a human health monitoring tool.

#### 4.1.6 Industry 6: energy

The Energy Industry Sector, including chemistry which is much influenced by the presence of IoT found, is labeled electricity, battery, gas, sensor gas and solar energy.

#### 4.1.7 Industry 7: mining

The mining industry sector which is much influenced by the existence of IoT, which is found is mining in the form of data mining labels, which is an advanced Information Technology (IT) industry.

There are several other industrial labels that are classified as widely used and are considered to have an IoT influence, namely logistics, IoT industry, supply chain management, decision support which are classified as operating management devices which in this study are categorized as all industries.

The manufacturing industry is the most mature sector in the application of the IoT concept with a large amount of research in this sector, this is reasonable because the 4th industrial revolution framework has emerged earlier and is very underlined about smart manufacturing, causing a lot of research to focus on this sector before moving to various industries others. Then the agriculture industry, researchers are very interested in this field because of the high opportunity for socio-economics, the rapid application of IoT techniques, and also land and plant objects involved in having very low applied risks. So that in terms of applied risk, very low risk is also the reason why the Public Service Industry is heavily influenced by IoT, as applies to the manufacturing sector. Then the electronics industry functions as a supporting tool that makes it easy for humans to access IoT services directly or IoT as a daily life support tool used by humans such as electric vehicles and wearable.

The passage of time, research has begun to shift a lot and reveal about the Hospital and Health care industry, even since 2016 this health topic is a new subject. Due to its application has a high risk because it is related to humans, but it can be interpreted that the level of IoT technology advancement has been quite mature since the time of the emerges at the time of the arrival of technology despite the very high level of risk to be applied. The future of research looks at the energy and chemistry industry as an important prospect in subsequent scientific research, this is not only a necessity because the more renewable energy, but also the presence of batteries is an important character of IoT energy availability to work so that ubiquitous can be easier to happen. Recent researchers are also very interested in conducting research related to data mining, the emergence of IoT which has resulted in the accumulation of very large data both offline and online into their own needs for analytic and then take advantage of the data for various interests of certain stakeholders.

### 4.2 IoT main character for industry

The development of IoT knowledge is very much and continues to be increasingly in demand by scientists and industry, it is because of the ability of IoT to connect many devices to be able to communicate with each other and enter information through the internet as delivered by (Kaur et al., 2018) even with a variety of different (heterogeneous) devices that can carry certain functions or benefits (Chan, 2015). Based on its function then IoT has a role, among others *identity, track* and *traceability*, and authentication (Liu et al., 2017), then *traceability* and *visibility* (Meng et al., 2017). Other perceptions that are not much different from industries perceptive (Zhang et al., 2018) outlining the benefits of IoT is to promote information progress in real-time *monitoring*, *traceability*, *tracking, transparency*, and *interaction*, which was then comprehensively elaborated by (Zhang et al., 2016) as *real-time traceability*, *visibility*, and *interoperability* in *production planning, execution, and control*.

This study found that there are four main things that are the main characteristics of IoT for industrial organizations (1) *Traceability*, (2) *Visibility*, (3) *Interoperability* and (4) *Interaction*. Whereas the role outside the industrial organization through the presence of IoT is included in green and eco-friendly process management (Al-Turjman, 2018), and IoT is also able to mitigate energy and green computing consumption (Mohiuddin and Almogren, 2019).

The development of IoT does not always bring positive things as discussed above, some negative things and problems are found related to the journey of IoT research and need to be considered by researchers including: technical or technological problems from IoT itself, industrial problems and businesses that require IoT solutions, problems in terms of IoT framework, problems when adopting and redesigning the current process, and problems after applying IoT. IoT design techniques have very poor communication latency problems and communication overhead (Jutila, 2016; Praveen Kumar and Babu, 2017; Al-Turjman, 2018) including the cost of remote wireless communication which is relatively expensive on some devices (Lv et al., 2016) which requires the right stage of device selection based on function and price. Including the limitations of the device in producing certain other variables needed such as "how much power is used" (Han et al., 2018). Overall the presence of the development of IoT is very fast, so in general, this IoT is difficult in terms of standardization as delivered (Tervonen, 2018). In order to achieve a good IoT system implementation (Fortino et al., 2017a) have a suggestion that developers may use IoT simulations based on either agent or network approach, which allows designers to validate their design choices and unfold unexpected conduct before the actual deployment.

#### 4.2.1 Problem came from the business needs that feasible be supported by IoT

• Agri-crisis occurs due to rain and poor climatic conditions, so farmers suffer from severe scarcity and have difficulty recovering from drought. IoT is a technology that serves as a solution to this problem (Srilakshmi et al., 2018). To do air quality tracking carried out only at the macro level, detailed hyper-local data is difficult to obtain because of the lack of sensors that collect information (Meinert et al., 2018). In addition, the relationship between weather, plants, and pests can negatively affect plant productivity and profitability (Moon et al., 2018)
• Problems in urban areas such as traffic jams, environmental contamination, limited natural resources (Paruchuri and Rajesh, 2018). Problems due to lack or absence of detection systems in the city (Díaz-díaz, Muñoz and Pérez-gonzález, 2017)
• Every authority (government) is required to know the pattern of citizen estimation for public service design and optimization goals, taken from a large number of passenger digital transaction records, this is the scope of researchers in IoT-driven urban computing applications (Weng et al., 2018)
• Changes in current manufacturing technology that require monitoring devices that support the distribution of sensors and high-speed wireless networks (Zhao et al., 2015), including manufacturing inspections in industry (Li et al., 2018b), and difficulties in planning of *production logistics* (PL) which increases due to uncertainty and dynamic production environment (Huang et al., 2019)
• Difficulty in building optimization that covers the entire production process (Liu et al., 2017) and how IoT can help activate the optimal composition of services (Li et al., 2018c)

##### 4.2.2 Problem came from the current IoT framework

• The framework is currently limited by the constraints of communication latency, fixed bandwidth, coverage, and uneven computing resources, therefore the framework is difficult to adapt to the emergence of IIoT demands or requirements (Li et al., 2018d).
• Not complete in calculating the factors that cause common problems that occur at this time in the system of *Production Logistic* - PL such as the following: distribution accuracy and low efficiency, lack of flexibility and responsiveness, and inconsistencies between distribution and production (Huang et al., 2019)
• IIoT is easy to hack and difficult to survive from various cyber-attacks (Falco et al., 2018)
• IoT has a large variety of devices, with different technologies and protocols. This brings its own disadvantages, so IoT is very low in terms of interoperability, security, scalability, efficiency or reliability (Trilles et al., 2017) including poor throughput running simultaneously (Al-Turjman, 2018)
• How to integrate production and logistics into a smart control system such as its ability to identify exceptions, self-organizing configurations, and self-adaptive collaboration (Zhang et al., 2018)
• Constant interactions that occur between machines, between humans, between humans and machines and the complexity of information from certain problems that arise will result in difficulties in exchange and sharing. Fundamental construction can lead to mutual understanding and awareness of the organizational information structure between users and agents (Hao et al., 2015).
• The superiority of IPv6 topology is also very concerned now to be used in implementing IoT in the future, where IPV6 can establish locative conditions on the Internet that are applicable and actually, due to IP distribution between objects that are not uniform at this time (Kleineberg and Helbing, 2017). In the IPv6 Internet topology, each node (device) represents the Autonomous System, this is a very perfect IoT concept to run.

##### 4.2.3 Problem came when adopting IoT and redesigning the current process

• Analyzing the current situation: before redesigning, the *reengineering* team needs to gain a better understanding of the chosen current process, paying attention to how badly it operates (bad or problematic processes), critical issues that affect performance, and a set of instructions for IoT adoption and redesign processes (Wolfs, 2017; Ammirato et al., 2018)
• Intensively understand ecological conditions at a fundamental level, the speed of communication, including information on certain distances to local servers (Keswani et al., 2018)
• How is worker *satisfaction* and how long the *adaptation* is needed to be competent in carrying out new activities (Ammirato et al., 2018)
• It is always difficult for designers to analyze and validate performance efficiently and effectively when it is associated with limited professional knowledge and there are *black-box* models (Zheng et al., 2018)

##### 4.2.4 Problem after IoT is applied

• With a manual approach to the amount of available system information and expert assumptions, it still poses difficulties in validating for experts to understand the system (Falco et al., 2018). Thus, the accumulation of data has a limitation, which presents a situation of large amounts of data but contains little information. So that *data mining* technology emerges; as an important tool in the acquisition of knowledge from the manufacturing environment (Liu et al., 2017)
• Applications must be able to find ways to obtain data (from the client side in it sensor-program code), to be stored reliably in large numbers and in a scalable way (digesting data in the database with all its difficulties), to transform that data into a which makes it possible to access them with *analytical* purposes then present them in a *dashboard* view (Lengyel et al., 2015)
• Sensors have poor sensory detectors, many physical parameters that cannot be detected, such as various things on dirty surfaces, mirrors or shadows, including positions that are slightly misaligned (Adelmann et al., 2006; Zhang et al., 2012)
• Various interconnected devices are difficult to maintain (*maintenance*) (Wolfs, 2017)
• Raw sensor data contains a lot of *noise*, is heterogeneous, and has high dimensions, which comes with a lot of complexity and computational difficulties in extracting high-quality results in a real-time manner (Vermesan and Friess, 2015)

### 4.3 A variety of research methods and tools, in the implementation of the concept of the internet of things

Researchers view IoT as an important research opportunity to solve various industrial problems, as the main character of IoT described in section B. Researcher can use several operational models in developing conventional industrial notation towards IoT services, which assist in verification and execution so that they can use the right tools as explained by (Fortino et al., 2017b). The growth of knowledge then combines science and technology into certain research methods that are applied through stages that focus on *defining one of the four layers of IoT technology* architecture as a domain of research knowledge: (1) the "Application and Service" layer, or (2) the "Platform" Architecture layer, also called data and knowledge, or (3) "Communication" Architecture layer, or (4) "Physical" Architecture layer called sensor or actuator (Meng et al., 2017), these are then processed to answer research questions three about what are the substance methods or tools used in the Internet of Things research (RQ3) in order to produce the substance methods or tools used in the IoT research.

**Fig. 6:**
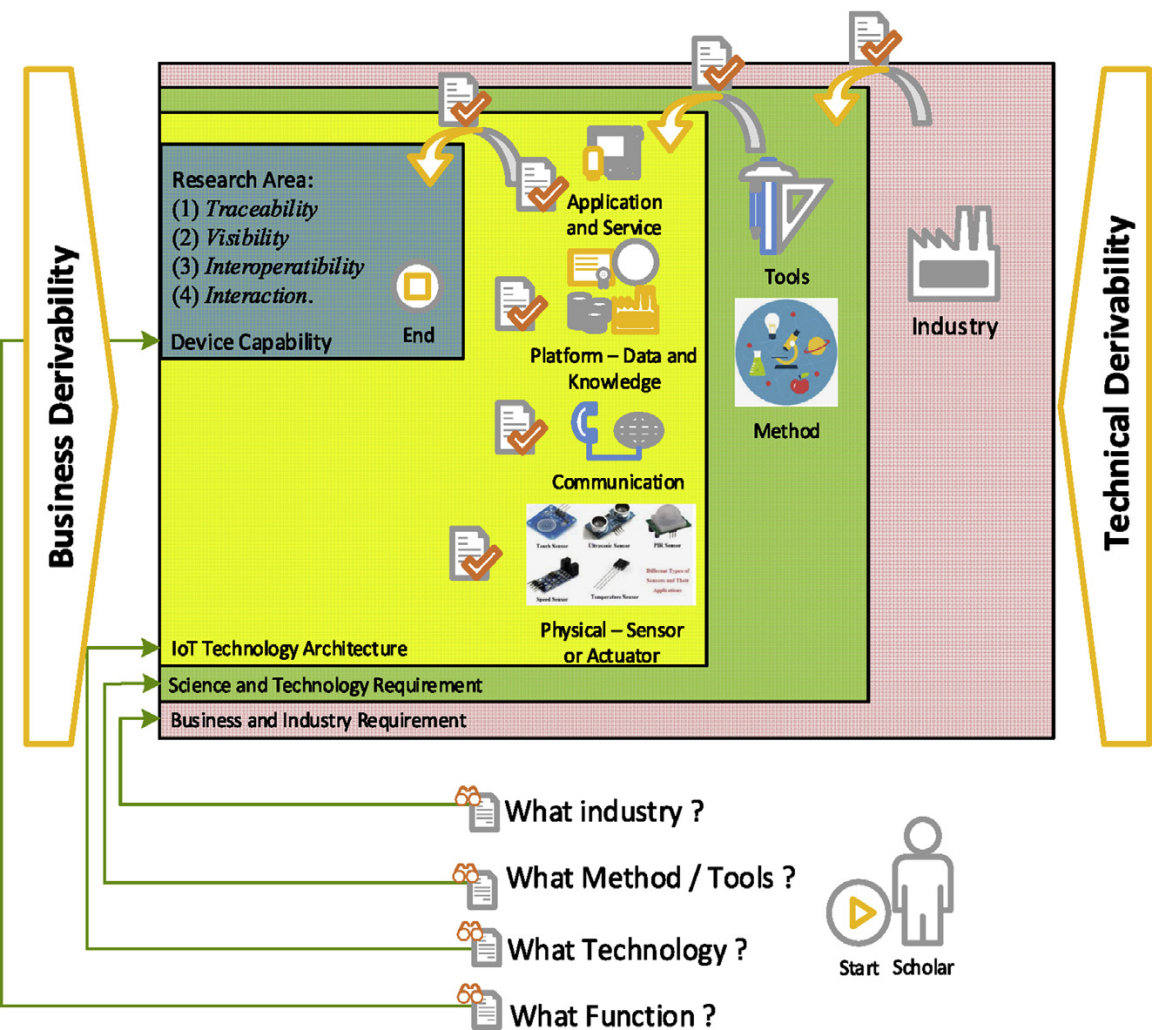
The IoT research thinking process.

All research begins with the phenomenon of business/industrial needs which are then faced with the willingness of IoT technology features so that they (researchers) look for novelty research with the help of methods based on appropriate science, as new discoveries into several context solutions, in the form of architecture own or combined technology, industrial technical application, or as a new context of knowledge from industrial management that is supported by IoT functions such as analysis of available real-time data. The researcher thinking process is shown in Fig. 6. The researcher processed the data through the bibliographic method for the entire 2006 IoT study until the end of 2018, obtained by finding IoT in the seven main industries in section A displayed in Tables 2, 3, and 4 where several terms (labels) were found that corresponded to each IoT technology used, described in each year the appearance of research. Through a comprehensive search of various studies with IoT keywords, industry and methods used by researchers; hence a variety of research methods are obtained which are considered to have a significant impact on research on the development of knowledge and strategic development and include impacts on the industry. Then these results are elaborated on the knowledge domain column according to emerging terms and components of basic IoT technology architecture which are ideas from the technical focus of research and development of science, as shown in Tables 5, 6, 7, 8, 9, and 10.

**Table 2:** Mapping research on technology architecture (2006–2015).

label	IoT Architecture Technology	Weight <Occurre nces>	Score <Avg. pub. year>
RFID technology	Physical	269	2014.2
RFID network	Physical	42	2014.6
Application system	Application	75	2014.7
Next generation network	Communication	37	2014.8
RFID system	Physical	233	2014.9
Low power wireless personal area networks	Communication	39	2014.9
Zigbee technology	Communication	40	2015.1
Service platform	Platform	178	2015.1
Passive RFID tag	Physical	39	2015.1
RFID reader	Physical	114	2015.3
Zigbee network	Communication	53	2015.3
WSN application	Application	46	2015.5
Actuator network	Communication	84	2015.5
Personal area network	Communication	78	2015.5
Sensor technology	Physical	178	2015.6
Open platform	Platform	41	2015.6
Middleware solution	Platform	72	2015.6
NFC	Communication	201	2015.7
Constrained application protocol	Application	144	2015.7
6LoWPAN network	Communication	72	2015.7
Zigbee	Communication	434	2015.7
Ad hoc network	Communication	102	2015.7
Network node	Communication	131	2015.8
Electric vehicle	Physical	96	2015.8
Middleware platform	Platform	68	2015.8
Wireless sensor network	Physical	2224	2015.8
Application development	Application	151	2015.8
Wireless sensor node	Physical	143	2015.9
Network environment	Communication	139	2015.9

**Table 3:** Mapping research on technology architecture (2016).

label	IoT Architecture Technology	Weight <Occurre nces>	Score <Avg. pub. year>
Global network	Communication	68	2016.0
e-Health application	Application	47	2016.0
Sensor system	Physical	127	2016.0
Wireless sensor networks	Physical	68	2016.0
Mobile ad hoc network	Communication	55	2016.0
Application programming interface	Application	56	2016.1
Constrained network	Communication	49	2016.1
Application developer	Application	91	2016.1
Network service	Communication	80	2016.1
Network topology	Communication	193	2016.1
Heterogeneous network	Communication	197	2016.1
IoT middleware	Platform	115	2016.1
GPS	Physical	209	2016.1
Wireless communication network	Communication	38	2016.1
Network coding	Communication	62	2016.1
Network architecture	Communication	333	2016.2
Network management	Communication	76	2016.2
Medical sensor	Physical	44	2016.2
Cloud database	Platform	40	2016.2
Network security	Communication	75	2016.2
Network size	Communication	35	2016.2
Network virtualization	Communication	45	2016.2
Sensor node	Physical	1141	2016.2
Application protocol	Application	130	2016.2
Information centric networking	Communication	109	2016.3
LTE network	Communication	69	2016.3
Network load	Communication	46	2016.3
Mobile network	Communication	246	2016.4
Body sensor network	Physical	53	2016.4
Web application	Application	158	2016.4
Vehicular network	Communication	59	2016.4
Lossy network	Communication	216	2016.4
Wireless local area network	Communication	54	2016.4
Medical application	Application	63	2016.4
Network device	Communication	95	2016.4
Access network	Communication	183	2016.5
Network performance	Communication	227	2016.5
Large scale network	Communication	56	2016.5
Wireless network	Communication	667	2016.5
Bluetooth	Communication	273	2016.5
Wireless mesh network	Communication	39	2016.5
Data networking	Communication	98	2016.5
Predictive analytic	Platform	40	2016.5
LTE	Communication	478	2016.5
Wi-Fi	Communication	303	2016.5
Vehicular ad hoc network	Communication	65	2016.6
Network parameter	Communication	37	2016.6
Network throughput	Communication	78	2016.6
Vehicle	Physical	1735	2016.6
Multiple sensor	Physical	73	2016.6
Mobile sensor	Physical	39	2016.6
Core network	Communication	77	2016.6
Home network	Communication	79	2016.6
Network congestion	Communication	60	2016.6
Mesh network	Communication	67	2016.6
Wearable sensor	Physical	98	2016.6
Wireless body area network	Communication	60	2016.7
Wi-Fi	Communication	313	2016.7
Bluetooth low energy	Communication	292	2016.7
Humidity sensor	Physical	57	2016.7
Cellular network	Communication	320	2016.7
Large network	Communication	33	2016.7
Application layer protocol	Application	62	2016.8
Network lifetime	Communication	273	2016.8
Network resource	Communication	139	2016.8
Android	Platform	171	2016.8
Smart city application	Application	137	2016.8
Diverse application	Application	46	2016.8
Sensor value	Physical	44	2016.8
Healthcare application	Application	135	2016.8
Camera	Physical	551	2016.8
Connected vehicle	Physical	53	2016.8
Temperature sensor	Physical	143	2016.9
Network capacity	Communication	64	2016.9
Network simulator	Communication	57	2016.9
Board	Physical	501	2016.9
Network traffic	Communication	161	2016.9
Sensors data	Physical	49	2016.9
Analytic	Platform	641	2016.9
Opportunistic network	Communication	49	2016.9
Big data analytic	Platform	210	2016.9
Surveillance camera	Physical	56	2016.9
Single board computer	Physical	73	2016.9
Analytical model	Platform	111	2016.9
IoT sensor node	Physical	38	2016.9

**Table 4:** Mapping research on technology architecture (2017).

label	IoT Architecture Technology	Weight <Occurre nces>	Score <Avg. pub. year>
Network bandwidth	Communication	60	2017.0
Network operator	Communication	84	2017.0
Analytical result	Platform	52	2017.0
Wearable	Physical	209	2017.0
Android application	Application	104	2017.0
Gas sensor	Physical	74	2017.0
Arduino board	Physical	54	2017.0
Software defined networking	Communication	113	2017.0
Motion sensor	Physical	41	2017.0
5G system	Communication	50	2017.0
Industrial IoT application	Application	34	2017.1
Things network	Communication	88	2017.1
Big data analytics	Platform	43	2017.1
IoT network	Communication	1208	2017.1
Network function	Communication	60	2017.1
Raspberry Pi	Physical	489	2017.1
Data analytic	Platform	298	2017.1
Arduino	Physical	156	2017.2
Wi-Fi network	Communication	47	2017.2
Biosensor	Physical	57	2017.2
Network condition	Communication	55	2017.2
5G technology	Communication	50	2017.2
Edge network	Communication	52	2017.2
Network edge	Communication	163	2017.2
Raspberry	Physical	38	2017.2
Wearable technology	Physical	59	2017.3
Sigfox	Communication	72	2017.3
5G network	Communication	209	2017.3
Ultrasonic sensor	Physical	53	2017.3
Network function virtualization	Communication	48	2017.3
Lora technology	Communication	34	2017.4
NB IoT system	Communication	53	2017.4
Unmanned aerial vehicle	Physical	79	2017.4
IoT applications	Application	150	2017.4
Low power wide area network	Communication	129	2017.4
Wi-Fi module	Communication	46	2017.5
Lora	Communication	326	2017.5
NB IoT	Communication	329	2017.5
Arduino uno	Physical	38	2017.5
Lora network	Communication	38	2017.5
Low power wide area networks	Communication	47	2017.5
Lorawan	Communication	191	2017.6
UAV	Physical	193	2017.6
PIR sensor	Physical	33	2017.7

**Table 5:** Growth of IoT knowledge on Manufacture.

Research	Domain Knowledge	Method/Tools	Industry
(Azeez et al., 2018)	Physical IoT	A resonance method based on square-patch antennas	Manufacture
(Huang et al., 2019)	Production Logistics optimization	Ant Colony algorithm	Manufacture
(Kumar, 2018)	Smart Manufacturing	Flexibility in systems, monitoring, and adaptation to changing needs	Manufacture
(Lee et al., 2018)	Smart Manufacturing	A light-weight Demand Response (DR) scheme, based on the Stackelberg Model	Manufacture
(Li et al., 2018b)	Smart industry	AI Method (Deep Learning) to detect the defects of the products	Manufacture
(Li et al., 2018c)	Cloud Manufacturing (CMfg)	EK-Oriented genetic algorithm (EK-GA) for the large-scale IoT service composition	Manufacture
(Liu et al., 2017)	Smart Manufacturing	IoT-enabled Intelligent Assembly System (IIASMP)	Manufacture
(Meng et al., 2018)	Adaptive Manufacturing	Manufacturing Reference Architectures (MRAs)	Manufacture
(Na et al., 2018)	Smart Manufacturing System	Maximum Weight Independent Set (MWIS)	Manufacture
(Riel et al., 2017)	smart products and digital manufacturing	Integrating functional safety and cybersecurity in the early design	Manufacture
(Rubaiee and Yildirim, 2018)	An energy-aware multiobjective on preemptive scheduling	Ant Colony algorithm	Manufacture
(Wang et al., 2018b)	Human-centered design factors for the design of interactive clothing	Kansei Evaluation method	Manufacture
(Wang and Hsieh, 2017)	Smart eyewear industry	Quality Function Development (QFD) to recognize the specific IoT development potential	Manufacture
(Xiao et al., 2018)	Fabrication of logic circuits	Bayesian Network (BN) method, and Probabilistic Transfer-Matrix (PTM) model	Manufacture
(Zhang et al., 2012)	Cloud Manufacturing	Fiber Bragg Grating perception network	Manufacture
(Zhang et al., 2018)	Mechanism and methodology of smart production	Smart Production Logistics Systems (SPLS)	Manufacture
(Zhang et al., 2016)	Real-Time Production Performance Analysis	Performance Analysis and Exception Diagnosis Model (PAEDM)	Manufacture
(Tsai and Lu, 2018)	Production planning and control	Activity-Based Costing (ABC) and Theory of Constraints (TOC)	Manufacture

**Table 6:** Growth of IoT knowledge of Agriculture.

Research	Domain Knowledge	Method/Tools	Industry
(Kang et al., 2018)	Smart Agriculture	agricultural cyber-physical-social system (CPSS)	Agriculture
(Keswani et al., 2018)	Smart irrigation control scheme	neural network	Agriculture
(Moon et al., 2018)	predictive weather on IoT platform.	lossy compression based on FWHT and DCT	Agriculture
(Nalajala et al., 2017)	Communication IoT	monitoring and control of greenhouse	Agriculture
(Patil and R, 2017)	Communication and platform IoT	Kalman filter (KF)	Agriculture
(Sawant et al., 2017)	Physical IoT	adaptation framework	Agriculture

**Table 7:** Growth of IoT knowledge on Public Service.

Research	Domain Knowledge	Method/Tools	Industry
(Al-Turjman, 2018)	Application and Service IoT	Hybrid Collaborative Path Finder (HCPF).	Public Service
(Naidu Are et al., 2018)	Application and Service IoT	Fire IoT sensors	Public Service
(Falco et al., 2018)	Communication IoT	automated attack generation based on artificial intelligence techniques	Public Service
(Jiang and Liu, 2018)	Application and Service IoT	solid waste transportation scheduling	Public Service
(Kong et al., 2018)	Application and Service IoT	parking space sharing and allocation problem	Public Service
(Weng et al., 2018)	passengers' closed transit chains	information enrichment and probabilistic inference	Public Service

**Table 8:** Growth of IoT knowledge on Electronics.

Research	Domain Knowledge	Method/Tools	Industry
(Chen et al., 2018)	Physical IoT	crumpled morphology onto the gold thin film using macro control	Electronics
(Erozan et al., 2018)	Physical IoT	electrolyte-gated field-effect transistors (EGFETs) based on inorganic materials	Electronics
(Kumar and Gandhi, 2017)	Communication IoT	transport layer security (TLS) protocol	Electronics
(Liu et al., 2018a)	Phisical IoT	flexible temperature sensors	Electronics
(Pyo et al., 2019)	Physical IoT	triboelectric nanogenerators (TENGs)	Electronics
(Razafimandimby et al., 2019)	Communication IoT	Neuro-Dominating Set algorithm (NDS)	Electronics
(Salmon and Meissner, 2015)	Phisical IoT	Monte Carlo simulations	Electronics

**Table 9:** Growth of IoT knowledge on Health.

Research	Domain Knowledge	Method/Tools	Industry
(Chai et al., 2018)	Application and Service IoT	Plan-Do-Study-Act method	Health
(Chukka and Kumar, 2018)	Application and Service IoT	IoT-Enabled ECG Telemetry system	Health
(Elhoseny et al., 2018)	Application and Service IoT	hybrid encryption schema	Health
(Kaur et al., 2018)	Application and Service IoT	vector machine and artificial neural network classifiers	Health
(Kim and Chung, 2017)	Application and Service IoT	knowledge-based crowdsourcing	Health
(Kim and Kim, 2018)	Application and Service IoT	Conjoint analysis	Health
(Li et al., 2018a)	Application and Service IoT	(a,k)-anonymity model	Health
(McRae et al., 2016)	Application and Service IoT	machine learning	Health
(Santhoshi and Thirugnanam, 2016)	Application and Service IoT	fall detection scheme using ambient sensors	Health
(Sarkar et al., 2017)	Application and Service IoT	blind cloud framework	Health
(Wang et al., 2018c)	Application and Service IoT	The Mann–Whiney test or t-test	Health
(Xie et al., 2018)	Platform IoT	knowledge in linked open data	Health

**Table 10:** Growth of IoT knowledge on Energy.

Research	Domain Knowledge	Method/Tools	Industry
(Bousdekis et al., 2018)	Application and Service IoT	“Detect-Predict-Decide-Act” proactivity principle	Energy
(Han et al., 2018)	Physical IoT	Ultra-low power (ULP) VLSI circuits	Energy
(Jutila, 2016)	Communication IoT	regressive admission control (REAC) and fuzzy weighted queueing (FWQ	Energy
(Liu et al., 2018b)	Physical IoT	tag searching	Energy
(Mohiuddin and Almogren, 2019)	Platform IoT	Workload-Aware Virtual Machine Consolidation Method (WAVMCM)	Energy
(Praveen Kumar Reddy and Rajasekhara Babu, 2017)	Physical IoT	Gravitational Search Algorithm (GSA) and Artificial Bee Colony (ABC) algorithm	Energy
(Reddy and Babu, 2017)	Communication IoT	Fuzzy C-Means (FCM) clustering algorithm	Energy
(Rizwan and Rajasekharababu, 2016)	Platform IoT	DVFS (Dynamic Voltage and Frequency Scaling) methods and existing effective optimal consolidation methods	Energy
(Sun et al., 2018)	Platform IoT	Markov decision process. Q-learning algorithm	Energy
(Wan et al., 2019)	Platform IoT	binary space partitioning (BSP)	Energy
(Li et al., 2018d)	Application and Service IoT	software-defined network (SDN) and edge computing (EC)	Energy
(Wang et al., 2018a)	Platform IoT	Offloading-assisted energy- balanced approach on IoT edge node relocation (CIC-OAEBA), and CIC-based Direct Replacement Approach (CIC-DRA).	Energy
(Zhang et al., 2018)	production and logistics	production and logistics	Energy
(Cui et al., 2018)	Physical IoT	PRG algorithm	Energy
(Ge et al., 2017)	Platform IoT	Forum Alert Traffic Security (FATS) architecture	Energy
(Gupta et al., no date)	Communication IoT	taxonomy of various solutions	Energy
(Hao et al., 2015)	Physical IoT	Agent-middleware technology	Energy
(Tervonen, 2018)	Physical IoT	Survey. business excellence and CogInfoCom	Energy

In traditional manufacturing industries, there are limitations to sensor technology, with many physical parameters unable to be detected, especially the need for long-term dynamic and real-time monitoring (Zhang et al., 2012). In today's modern industry there are several new terms that describe future industry concepts, such as Manufacturing 2.0, Internet Industry, Smart Factory, 4th industrial revolution and IIoT (Riel et al., 2017; Meng et al., 2018).

The broad topic of IoT research for 4th industrial revolution in the manufacturing area as mentioned in Table 5 is about connecting all components in manufacturing systems using various sensor systems, Cyber-Physical Systems (CPS) through the IoT concept, where activity data from all components can be real-time collected and monitored, to provide a smart response to various problems that may arise in the factory, including the results of real-time analysis obtained from cloud computing and big data (Tsai and Lu, 2018).

Specific IoT research topics for 4th industrial revolution provide opportunities for smart manufacturing in terms of real-time traceability, visibility and interoperability in production planning, implementation and control (Zhang et al., 2016), flexibility in systems, monitoring, and adaptation to change manufacturing needs (Kumar, 2018), besides that reliability is also an important research topic in other IoT applications and cloud environments (Xiao et al., 2018).

IoT implementation is considered to provide various benefits in supporting manufacturing operation's internal processes or activities, such as optimization of production logistics in utilizing the real-time data generated by the IoT (Huang et al., 2019). Other benefits include smart energy management that significantly saves operational expenses and minimizes total product completion time (Rubaiee and Yildirim, 2018), detect product defects (Li et al., 2018b), ultimately increasing profitability and production efficiency (Zhang et al., 2018). In terms of R & D operations, IoT technology is able to bridge the gap between humans and technology that can be used for interactive innovation (Wang and Hsieh, 2017).

In terms of technical application, research (Liu et al., 2017) provides an important framework for companies that already have high technology potential and then want to activate IoT, through IoT-enabled intelligent assembly systems for mechanical products (IIASMP), through their research questions as following: (1). How to encode current manufacturing resources, through data parsing, exchange, processing, and sharing? (2). How to capture massive data from heterogeneous devices then transfer and integrate it? (3). What methodology is appropriate for value-added information for the company's management decision-making process? (4). How to achieve optimization from the current manufacturing process.

Research in the agricultural area is largely directed at analyzing sensor utilization through the Wireless Sensor Network (WSN) system which is actually not IoT, even though this WSN application is part of building an IoT solution. WSN research is conducted throughout the World for precision agricultural purposes (Sawant et al., 2017), requiring many improvements in the fields of communication, data distribution, and real-time component analysis to make dynamic decisions. For example, IoT wireless sensor environment development that is able to accurately analyze soil and environmental parameters used in agricultural activities to predict air demand in a timely manner (Keswani et al., 2018), including management of agricultural production, with case studies in solar greenhouses (Kang et al., 2018).

Other research uses the concept of IoT through smarter, more complex farming, looking at opportunities through plant and land data supported by sensors, so that embedded sensors are expected to be used for crop yield prediction, crop classification, soil classification, weather prediction, and crop prediction with using decision-making systems on existing IoT components such as the IoT Gateway and IoT Service platforms integrated in the system to provide smart plant growth solutions for farmers (Patil and R, 2017), other studies focus on diverse agricultural data that need to be stored efficiently and beneficial (Moon et al., 2018).

Research on IoT applications for public services is currently associated with the usual use of the Internet to communicate with other devices to achieve certain benefits in urban areas, this is supported by the development of today's critical infrastructure that is 'smarter' and more dependent on highly specialized computers called industrial control system (ICS) (Falco et al., 2018). Benefits are obtained through the use of approval, sensing and information functions for everyday human activities. According to (Jiang and Liu, 2018), research on the IoT area can be carried out on the following three main aspects (1) How to recognize data transmission objects and technology. (2) Data communication technology, which is about how to act on data and technology. (3) Understanding and adaptation of historical data of IoT device users based on data and reasoning.

Recognizing objects and data transmission technology in the concept of public services is closely related to infrastructure and how to control it, such as CCTV, electricity network, air network and transportation network security (Falco et al., 2018), real-time garbage collection scheduling based on certain conditions (Jiang and Liu, 2018), management of public parking spaces by developing urban parking management cloud platforms (Kong et al., 2018), fire security and monitoring (Naidu Are et al., 2018).

Understanding data transmission communication technology in the concept of public communication, for example (Al-Turjman, 2018) that uses IoT hybrid sensing communication for smart cities, which facilitates the involvement of heterogeneous traffic flows in network sensors so that it can be used for simultaneous users with various needs.

Regarding the understanding and adapting the historical data for IoT device users, for example the Intelligent/Smart and Connected Transportation System (ICTS) understands the preferences and demands of actual passenger behavior collected passively from IoT devices to reduce passenger transit chains using information enrichment and probabilistic inference approaches (Weng et al., 2018).

Growth of IoT knowledge on Electronics, As a result of the use of IoT which requires sensor networks, causing dramatically increasing the rapid growth of portable electronics. The growing popularity of various sensors and portable devices, causing the demand for electricity to drive these electronic devices is very important. Although there is also research in the era of security on IoT devices in the information transport layer such as (Kumar and Gandhi, 2017; Erozan et al., 2018).

Many of research in the electronic field has been investigated regarding energy harvesting against high output performance, which is one of the most important barriers to practical application (Chen et al., 2018) because powering electronics is a big challenge using battery technology with power limited. As research is conducted on circuit design methods to improve the efficiency of charging to energy storage devices (Pyo et al., 2019), then triboelectric nanogenerator (TENG) research based on triboelectrification and electrostatic induction make energy harvesting technology simple, cost-effective, and versatile (Liu et al., 2018a).

There is also a flexible Electronic concept, which requires information to be distributed on whatever surface we need, where development is highly demanded the IoT, this case associated with robot technology and electronic skin (Liu et al., 2018a). Recent research is now beginning to shift, from the IoT paradigm to just being passive so far, to then add an active role to IoT devices using robots. ABI research introduces a new concept called Internet of Robotic Things (IoRT) as a set of intelligent devices that can monitor events, integrate sensor data from various sources, use local intelligence and then distribute it to determine the best actions, ultimately acting to control or manipulate objects in physical world (Razafimandimby et al., 2019). An interesting vision for the IoT is a group of flying robots (robot bees) that can provide enormous collective intelligence to gather information. However, this vision cannot be implemented because it requires a new method of providing a new architecture to combine primitive information into collective intelligence (Salmon and Meissner, 2015).

The research on the Health area, there is a paradigm shift from traditional and IoT-based medical field care, where doctors now have to pay more attention to the patient's raw medical records, then directly in making medical advice, conclusions or diagnoses from their experience using the hospital information system (HIS). The IoT-based HIS is distributed by scattered devices such as tablet computers, personal digital assistants that are used as automatic analyzers, or other massive and informative medical devices (Xie et al., 2018) and the possibility of a diagnosis can be made by a doctor elsewhere when the doctor has time for special patients, so the diagnosis can be more precise.

IoT research in the health sector, it is important to pay attention to various attributes of health information such as the service provider profession, task discussion room, devices used, expert support, and various personal medical data (Kim and Kim, 2018). Various medical data can be obtained by utilizing IoT devices that are in our daily lives, such as jam, health tape, scale, TV, lights, and door lock (Kaur et al., 2018), or various types of related sensors used such as smart accelerometers, gyroscope, pulse oximeter, RBG camera, camera Kinect, micro cellphone, PIR, RFID, smart tiles, etc., where these sensors are integrated with IoT technology (Santhoshi and Thirugnanam, 2016) and all these sensors can support, personal health records and self-diagnoses (Kim and Chung, 2017), for example, IoT to monitor human physiological conditions indefinitely, so doctors examine their patients remotely such as monitoring using brain safety research obtained through sensors (Kaur et al., 2018).

IoT research in the energy sector largely relies on energy efficiency that can be applied to a wide variety of fields with the benefits brought by IoT, both in internal IoT devices that require energy to run (usually batteries or solar panels), as well as external use of resources large-scale energy such as power-networks that are helped by IoT in reducing unnecessary or inefficient energy. The basic idea of the various studies is IoT that is able to complement network connectivity and computing capabilities to various physical devices.

Smart-grid is a sub-function of the smart city today, its research has become an evolution in managing electricity demand that is large to be managed, smart and economical, in the Heterogeneous Network infrastructure. Smart-grid can automatically support electricity in the city and adjust to changes in user demand. This is supported by IoT where electricity meters are connected to the Internet to provide consumers/suppliers with smart decisions about energy use/production in real time. For example, smart home appliances (dishwashers, clothes, and air conditioners) communicate over the network using smart meters and electric machines to avoid peak times (Al-Turjman, 2018).

The idea of IoT research then continues to develop through the integration of various infrastructure attributes such as hospitals, electricity networks, energy, transportation, food, air, etc. The idea of the researcher (Al-Turjman, 2018) with the “Agile IoT” method uses middleware signal processing to allow sensors in the infrastructure to be redesigned by applying ideas 1) Trying parallel communication methods, and 2) Feeling parallel mechanical parameters, to utilize the temperature sensor parameters for non-temperature measurements, such as fluid flow in a pipe (air, plant), ice buildup (transportation, energy, manufacturing), and mechanical doors (medicine storage cabinets in hospitals). “Green computing” method for energy use efficiency, where virtual machines replace idle physical servers into hibernation mode, thereby reducing power usage (Mohiuddin and Almogren, 2019).

Energy research in its own IoT internal devices, power consumption is a major concern for on-chip system designers, such as using ultra-low power VLSI (ULP) circuits that have benefited greatly from academia and industry as the most suitable technique for IoT devices (Han et al., 2018), a method with a combination of Optimal Secured Energy Conscious Protocol (OSEAP) and Improved Bacterial Foraging Optimization (IBFO) algorithm (Praveen Kumar Reddy and Rajasekhara Babu, 2017), resource allocation using the DVFS (Dynamic Voltage and Frequency Scaling) method and method optimal effective consolidation (Rizwan and Rajasekharababu, 2016).

### 4.4 The thinking process on how scientists formulate IoT research

According to the systematic research survey result, it can be assumed there is a logically consistent of the scholar thinking process such framed in Fig. 6.

The main research idea based on the business domain or technical derivability, which are crossed over among ideas. Based on the deductive way, a researcher can begin the process of sequential research thinking starting from the point of view of business and industrial needs as a broad-scale need. Then leading to the need for science and technology using certain tools and methods. Then based on IoT technology architecture that focuses on one or more applications, platforms, communications, and sensors. In the end, research involving the IoT system utilizes the main capabilities of each IoT device that has traces, views, operations and facilitates something.

## 5 Conclusion

Paper made an important contribution, regarding the growth and development of the IoT in the following stages: First, applying a new research approach to all Scopus literature, reliable paper journals and proceedings from the beginning of 2006 until the end of 2018 without exception, as a dynamic representation of the growth of knowledge in the field to what are the main component themes. Second, exploring the field of the IoT with industry classification offers a comprehensive review. The combination of co-citation offers a perspective on the work of the most influential and most productive industries in the IoT. Third, this paper looks at Growth and Development of the IoT in Industry, about evaluating the problems that exist in defining needs, a technology that can be helped by the presence of IoT, problems when implementing IoT technology, until after the implementation of IoT. Fourth, this paper reaches out to thinking patterns from existing forms of research, by framing the thinking process of researchers.

This paper represents an overview of the field of IoT by combining two important perspectives - observing evolution and observing IoT directly on the most prominent themes in the Industrial sector and also as an accurate foundation for seeing new IoT business opportunities and research opportunity.

The most important factor that determines what is the most influential theme for researchers in the IoT found in this study are (1) the emergence of the 4th industrial revolution as an interesting topic for researchers because it has many novelties especially in large-scale industrial reforms manufacture, then (2) the broad opportunities and benefits of socio-economic research as shown by agriculture results in the result and analysis section A, and (3) considering how the level of risk from applying the research, such as those found in Hospital and healthcare industries which requires time for technological readiness.

Based on the perspective of researchers, how to start new research on IoT is how they need to focus on research gaps based on certain phenomena, but their research must be built on concepts about industry, methods, tools, technology, and functions as a deductive-scientific approach. Research can also participate in following the trend that moves over time, where since the last two years research on hospitals, healthcare and energy has been widely researched and indicates that there will be many novels in this field.

There is a dependence on the time span of the dataset being studied, which allows for replication of the analysis in the future, at the same time period or in between, while the analysis of the next timeframe will produce different outcome lines. However, the overall study only processes Scopus indexed papers. There is a possibility that some papers that have significant scientific knowledge are excluded in research surveys, such as white paper from consulting firms, research companies, and government institutions, and IoT vendor companies. Therefore, this paper provides a good starting point for providing business solutions as well as dynamic literature reviews that are similar to other fields, or some explorations that are sub-sections of the IoT, for example, the results obtained can be further analyzed for positive roles and IoT negative in its application in the industry, what methods/tools are widely used, and so on.

## Declarations

### Author contribution statement

Muhammad Dachyar: Conceived and designed the experiments; Analyzed and interpreted the data.

Teuku Yuri M. Zagloel: Performed the experiments.

Lihardo Ranjaliba Saragih: Contributed reagents, materials, analysis tools or data; Wrote the paper.

### Funding statement

This work was supported by the University of Indonesia in the "TaDok 2019″ grant scheme.

### Competing interest statement

The authors declare no conflict of interest.

### Additional information

No additional information is available for this paper.
